# Cycling Cross-Bridges Contribute to Thin Filament Activation in Human Slow-Twitch Fibers

**DOI:** 10.3389/fphys.2020.00144

**Published:** 2020-03-24

**Authors:** Alfredo Jesus López-Dávila, Joseph M. Chalovich, Stefan Zittrich, Birgit Piep, Faramarz Matinmehr, Andras Málnási-Csizmadia, Anna Á. Rauscher, Theresia Kraft, Bernhard Brenner, Robert Stehle

**Affiliations:** ^1^Institute of Molecular and Cell Physiology, Hannover Medical School, Hanover, Germany; ^2^Department of Biochemistry and Molecular Biology, Brody School of Medicine, East Carolina University, Greenville, NC, United States; ^3^Institute of Vegetative Physiology, University of Cologne, Cologne, Germany; ^4^MTA-ELTE Motor Pharmacology Research Group, Department of Biochemistry, Eötvös Loránd University, Budapest, Hungary; ^5^Optopharma Ltd., Budapest, Hungary

**Keywords:** slow-twitch muscle, human striated muscle, skinned fibers, thin filament, troponin, thick filament, myosin, regulation of muscle contraction

## Abstract

It has been shown that not only calcium but also strong binding myosin heads contribute to thin filament activation in isometrically contracting animal fast-twitch and cardiac muscle preparations. This behavior has not been studied in human muscle fibers or animal slow-twitch fibers. Human slow-twitch fibers are interesting since they contain the same myosin heavy chain isoform as the human heart. To explore myosin-induced activation of the thin filament in isometrically contracting human slow-twitch fibers, the endogenous troponin complex was exchanged for a well-characterized fast-twitch skeletal troponin complex labeled with the fluorescent dye N-((2-(Iodoacetoxy)ethyl)-N-methyl)amino-7-nitrobenz-2-oxa-1,3-diazole (fsTn-IANBD). The exchange was ≈70% complete (*n* = 8). The relative contributions of calcium and strong binding cross-bridges to thin filament activation were dissected by increasing the concentration of calcium from relaxing (pCa 7.5) to saturating levels (pCa 4.5) before and after incubating the exchanged fibers in the myosin inhibitor para-aminoblebbistatin (AmBleb). At pCa 4.5, the relative contributions of calcium and strong binding cross-bridges to thin filament activation were ≈69 and ≈31%, respectively. Additionally, switching from isometric to isotonic contraction at pCa 4.5 revealed that strong binding cross-bridges contributed ≈29% to thin filament activation (i.e., virtually the same magnitude obtained with AmBleb). Thus, we showed through two different approaches that lowering the number of strong binding cross-bridges, at saturating calcium, significantly reduced the activation of the thin filament in human slow-twitch fibers. The contribution of myosin to activation resembled that which was previously reported in rat cardiac and rabbit fast-twitch muscle preparations. This method could be applied to slow-twitch human fibers obtained from the soleus muscle of cardiomyopathy patients. Such studies could lead to a better understanding of the effect of point mutations of the cardiac myosin head on the regulation of muscle contraction and could lead to better management by pharmacological approaches.

## Introduction

Mammalian muscles are either smooth or striated. Striated muscles are further classified as cardiac, fast-twitch skeletal, and slow-twitch skeletal. Each type of muscle has properties suited to its function. Activation of all striated muscles are triggered by increases in intracellular calcium concentration but the mechanism of regulation and contribution of myosin to activation differs among the muscle types (Gordon et al., 2000; Schiaffino and Reggiani, 2011; Shadrin et al., 2016). These differences in mechanism provide us with the possibility of selectively altering the activity of a particular muscle type in order to treat disease. One incompletely resolved issue of regulation is the role that actively cycling cross-bridges play in activation of the thin filament in striated muscle, particularly in slow-twitch and in human muscles.

It is clear in solution studies that myosin is both an enzyme and an activator of the regulatory apparatus of striated muscle (Eisenberg and Weihing, 1970; Bremel et al., 1973; Houmeida et al., 2010; Baxley et al., 2017; Johnson et al., 2018). The activating ability of myosin depends on the nucleotide bound to myosin but is somewhat independent of the binding affinity (Chalovich et al., 1983). Examples of activating myosin species are those lacking bound nucleotide or those containing bound ADP. Several models of regulation include both calcium and rigor type myosin binding as activators (Hill et al., 1981; McKillop and Geeves, 1993).

Activation of actin thin filaments by myosin heads has also been observed in isometrically contracting skinned fast-twitch muscle fibers. In this preparation, myosin-induced activation was shown at submaximal calcium concentrations after exchanging the endogenous fast-twitch skeletal troponin complex (fsTn) for an fsTn labeled with N-((2-(Iodoacetoxy)ethyl)-N-methyl)amino-7-nitrobenz-2-oxa-1,3-diazole (IANBD) on Cys 133 of troponin I [IANBD-labeled fsTn (fsTn-IANBD)] (Brenner and Chalovich, 1999; Brenner et al., 1999). Myosin-induced activation was also observed at saturating calcium concentrations using other probes (Sun et al., 2006; Fusi et al., 2014).

Cycling cross-bridges also activate cardiac muscle thin filaments at submaximal and saturating calcium levels. For example, lowering “strong” cross-bridge binding with orthovanadate reduced both calcium sensitivity and activation in isometrically contracting rat papillary muscle bundles (Rieck et al., 2013; Li et al., 2014). Similarly, reducing the number of force producing cross-bridges with blebbistatin reduced both the calcium sensitivity and the total activation of the thin filament (Robertson et al., 2015; Kampourakis et al., 2016, 2018; Zhang et al., 2017). On the other hand, both myosin regulatory light chain phosphorylation and treatment with omecamtive mecarbil (to increase the population of force producing cross-bridges) increased calcium sensitivity of activation in isometrically contracting rat cardiac trabeculae (Kampourakis et al., 2016, 2018). However, at least one study reported no activating effect of cycling cross-bridges in this preparation (Sun et al., 2009).

Little information is available on the contribution of cycling cross-bridges to thin filament activation in slow-twitch human muscle fibers. Because human slow-twitch fibers contain the same myosin heavy chain isoform as the human heart muscle (Gordon et al., 2000; Sheng and Jin, 2014) such information could be of great use. Slow-twitch fibers from soleus muscle biopsies of carriers and patients suffering myosin-based, inherited cardiomyopathies are an established *ex vivo* model for studying the disease mechanism in humans (Kohler et al., 2002; Kirschner et al., 2005; Seebohm et al., 2009). For these reasons, we investigated myosin-induced thin filament activation in isometrically contracting human slow-twitch soleus muscle fibers.

To probe the activation level of the thin filament, we exchanged endogenous slow-twitch skeletal troponin (ssTn) for fsTn-IANBD. Cys 133 is only present in fsTn, and it provides a convenient location for attachment of an environmentally sensitive probe (Trybus and Taylor, 1980; Brenner et al., 1999; Lopez-Davila et al., 2012). This label is able to detect the activating effect of myosin on the thin filament as previously reported (Trybus and Taylor, 1980; Brenner and Chalovich, 1999; Brenner et al., 1999). We used this well-established sensor of the thin filament activation in our human soleus muscle fibers in order to determine a possible activating effect of cycling cross-bridges during isometric contraction in slow-twitch skeletal muscle. We analyze the implications of this isoform exchange for our results in the last sections of the discussion.

Our results suggest that not only calcium but also strongly bound, cycling cross-bridges contribute to the thin filament activation state of slow-twitch human soleus muscle fibers. Thus, in spite of having a fast-twitch troponin complex as a sensor, our slow-twitch fibers are able to detect the activating effect of the human cardiac myosin heavy chain on the human thin filament during isometric contraction, as previously reported in rat trabecula. The observation of the strong activating effect of cycling cross-bridges on thin filaments in isometrically contracting human slow-twitch fibers has implications in understanding the crosstalk between thin and thick filaments, the development of myosin-based cardiomyopathies, and their therapeutic treatment with small molecules.

## Materials and Methods

### Preparation of Troponin

Purification of the rabbit fsTn consisting of the three fast-twitch rabbit subunits and its labeling with IANBD on Cys 133 of troponin I were accomplished as previously described (Lopez-Davila et al., 2012).

### Preparation of Fibers

Biopsies of the soleus muscle were obtained from healthy adult individuals and stored as for previous studies (Kohler et al., 2002; Kirschner et al., 2005). Approval of Hannover Medical School Ethics Committee was obtained for use of anonymous muscle biopsies. All subjects gave written informed consent in accordance with the Declaration of Helsinki. Single slow-twitch fibers were prepared from the biopsies according to the procedure originally described for psoas muscle fibers (Yu and Brenner, 1989).

### Preparation of Solutions

The exchange, rigor, pre-rigor, relaxing, and activating solutions were used as described for fast-twitch skeletal muscle (Brenner and Chalovich, 1999). The compositions of solutions (all adjusted to pH 7.0 at 20°C) are given in the Supplementary Material. Free calcium concentrations in experimental solutions were expressed as pCa (log[Ca2^+^]/M) and were in the range of 7.5 (relaxing solution) to 4.5 (activating solution).

### Inhibition of Force Generation by Para-Aminoblebbistatin (AmBleb)

AmBleb, a recently developed derivative of blebbistatin (Varkuti et al., 2016), was used to inhibit myosin heads from entering the strong binding, force-generating states. Blebbistatin and its derivatives stabilize the closed state of the switch 2 element of the nucleotide binding site (Zhao et al., 2008; Xu et al., 2009). In this state, in which both ADP and P_*i*_ are bound to the active site, formation of the strong actomyosin interaction is inhibited (Rauscher et al., 2018). We used AmBleb since the regular blebbistatin has an auto emission spectrum that overlaps with the emission spectrum of IANBD. AmBleb has additional advantages of being non-fluorescent, photostable, non-cytotoxic, and non-phototoxic, while its solubility is more than 40 times higher than that of blebbistatin (Varkuti et al., 2016). The concentration of AmBleb was determined using its extinction coefficient at 427 nm of 6100 M^–1^cm^–1^ in DMSO. The effective concentration of AmBleb required to inhibit most of the active force generation was determined prior to analyses with fsTn-IANBD containing fibers.

### Recording of Force and IANBD Emission Intensity and Experimental Protocol

The experimental apparatus for recording force, sarcomere length (SL), and IANBD emission intensity was previously described (Brenner and Chalovich, 1999; Brenner et al., 1999). SL was measured by laser diffraction (Brenner, 1983) of the same fiber segment (2.5 mm in length) used for the excitation and emission of IANBD. IANBD fluorescence was excited using a mercury arc lamp (Scientific Instruments Heidelberg GmbH. Heidelberg, Germany) and a 450/70 nm bandpass interference filter. IANBD emission was measured using a 535/40-nm bandpass interference filter. IANBD fluorescence depends linearly on the number of fsTn-IANBD molecules in the field of vision. That number is directly proportional to the number of sarcomeres and inversely proportional to the SL in the focal area. Therefore, IANBD fluorescence was multiplied by the SL to normalize fluorescence to the amount of fsTn-IANBD. The rate constant of isometric force redevelopment after a short period of isotonic shortening *k*_tr_ was measured as previously described (Brenner, 1983; Brenner and Eisenberg, 1986).

Single-skinned fibers (8–10 mm in length) were attached between the force transducer and a lever system of the apparatus using cyanoacrylate glue (Histoacryl; B. Braun Surgical GmbH. Melsungen, Germany). The ends of the fibers were stiffened with glutaraldehyde as previously described (Kraft et al., 1995). Prior to activation, the SL was set to 2.3–2.4 μm in relaxing solution. The fiber was transferred to activating solution, and isometric force was recorded. Subsequently, the endogenous ssTn was exchanged for the fsTn-IANBD. For the exchange procedure, fibers were first transferred to pre-rigor solution for 1–2 min and then to rigor solution for 30 min to remove any trace of ATP. The fibers were then transferred into exchange buffer containing 0.5 mg/mL fsTn-IANBD. After an exchange period of 3.5 h, excess, unbound fsTn-IANBD was removed by washing the fibers for 30 min in rigor solution. IANBD emission intensity and mechanical parameters were then measured under two experimental conditions. First, the pCa–force relationship and pCa-IANBD emission intensity relationship were recorded simultaneously by exposing the fibers to submaximal and maximal calcium concentrations. Second, the same fiber was incubated for 10 min in relaxing solution containing 50 μM AmBleb in order to avoid a possible activating effect of the myosin heads on the thin filament. Then, the pCa–force relationship and IANBD emission intensity were simultaneously recorded again. Because AmBleb absorbs light at the excitation and emission wavelengths, it was necessary to omit AmBleb during the brief time of measurement of force and IANBD fluorescence. Thus, we used a two-chamber protocol: The fiber was placed for a short period of time in an acquisition chamber containing the activating buffers and then back in a non-acquisition chamber containing relaxing solution and 50 μM AmBleb. This AmBleb re-incubation protocol avoids a slow, cumulative loss of the AmBleb effect on the fiber during this part of the experiment. In both parts of the experiment, fiber striation was stabilized during calcium-activation by permanently cycling the fiber every 5 s between isometric steady state contraction and short (330 ms) periods of lightly loaded (about 5% of maximal force) isotonic shortening with subsequent re-stretch to the initial (isometric) SL (Brenner, 1983). After completing both parts of the experiment, the fiber was stored at −20°C for subsequent quantification of the amount of troponin exchange by PAGE-Western blot analysis (see Supplementary Material).

### Confocal Microscopy

The duration of the troponin exchange period was established from a separate set of skinned fibers in which the time response of the troponin exchange was followed using a confocal fluorescence microscopy system coupled to an inverted microscope placed under the mechanical setup as previously described (Kraft et al., 1995). In short, specimens were excited at 488 nm, and emission detected at 517/40 nm. Uniform distribution of the labeled troponin was quantified by means of the ratio of the intensity at superficial areas over intensity in the core, as seen in longitudinal (optical) sections through the center of the fibers.

### Data Analysis

Force and fluorescence transients were fitted by a mono-exponential function in biokine 32 (version V4.27, Biologic). The steady state dependence of force and emission intensity of IANBD on calcium concentration was fitted to data from individual fibers using the Hill equation. All obtained parameters were analyzed in GraphPad Prism (version 4.02). All values are given as mean ± SEM with n representing the number of experiments. Mean values were compared using paired Student’s *T*-test (*p* < 0.05) in GraphPad Prism (version 4.02).

## Results

### Homogeneity and Amount of Troponin Exchange

Essential prerequisites for analyzing thin filament regulation in contracting muscle fibers under meaningful conditions are large extent of exchange, specificity of exchange, and preservation of functional and structural viability of the fsTn-IANBD exchanged slow-twitch muscle fibers. These issues were addressed by confocal imaging, PAGE-Western blot analysis, and characterization of mechanical properties.

**FIGURE 1:**
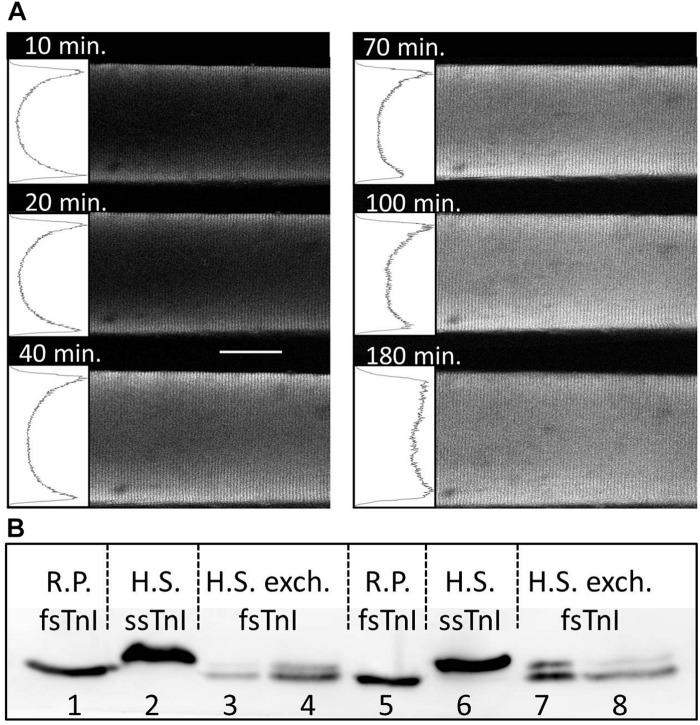
Exchange of native slow-twitch skeletal troponin complex (ssTn) complex for IANBD-labeled fsTn (fsTn-IANBD) complex. **(A)** Spatial and temporal equilibration of fsTn-IANBD within the cross-section of a single slow-twitch human fiber imaged by confocal microscopy. Clear insets at the left of each picture represent mean intensity profiles in transverse direction of the fiber that were integrated over a longitudinal length of 20–25 sarcomeres. Sarcomere length is 2.4 μm. White bar: 50 μm. **(B)** Western blot analysis showing TnI from single skeletal muscle fibers. Lanes 1 and 5 show the TnI content in single rabbit fast-twitch psoas muscle fibers [R.P. fast-twitch skeletal troponin I subunit (fsTnI)], and lanes 2 and 6 the TnI content from single human slow-twitch soleus muscle fibers [H.S. slow skeletal troponin I subunit (ssTnI)]. Lanes 3, 4, 7, and 8 show the TnI content from single human slow-twitch soleus muscle fibers after 3.5 h incubation in 0.5 mg/mL fsTn-IANBD and performing the mechanical experiments. Note that after the troponin exchange procedure ssTnI is reduced and fsTnI is present in the human slow-twitch fibers, confirming the exchange of ssTn by fsTn-IANBD.

The spatial and temporal course of the equilibration of fsTn-IANBD within the cross-section of a fiber is shown in Figure 1A. At 10–100 min of incubation in 0.5 mg/mL fsTn-IANBD, IANBD emission intensity was lower at the core than at the edges, suggesting incomplete troponin exchange in the core. The relative intensity in the core increased with incubation time up to 3 h, whereupon it remained stable (see the insets at the left of every picture in Figure 1A and Supplementary Figure S1 showing the core/edge ratio of those mean intensity profiles at different incubation times). The slightly lower emission intensity at the core of the fiber after 3-h incubation could be due to absorption of exciting and emitted light in the *z*-axis direction. Therefore, we incubated the fibers for 3.5 h in 0.5 mg/mL fsTn-IANBD before doing mechanical experiments. The striated pattern of emission observed in the Figure 1A was in line with previous studies showing that fsTn-IANBD specifically bound to the overlap region of sarcomeres where the rigor cross-bridges activate the thin filament (Brenner et al., 1999; She et al., 2000; Lopez-Davila et al., 2012).

Figure 1B shows Western blot analysis of troponin I subunits (TnI) in single muscle fibers. After incubation in 0.5 mg/mL fsTn-IANBD, human slow-twitch fibers contained not only the endogenous slow skeletal troponin I subunit (ssTnI) but also a high amount (67.7 ± 4.8%, *n* = 8) of fast skeletal troponin I subunit (fsTnI). The presence of fsTnI in slow-twitch muscle fibers confirms the effective exchange of fsTn-IANBD. This amount of exchange was similar to previous reports (Bell et al., 2006; Solzin et al., 2007; Neulen et al., 2009; Wijnker et al., 2014).

### Effects of Troponin Exchange and of the Experimental Protocol on Functional Properties of Fibers

**FIGURE 2:**
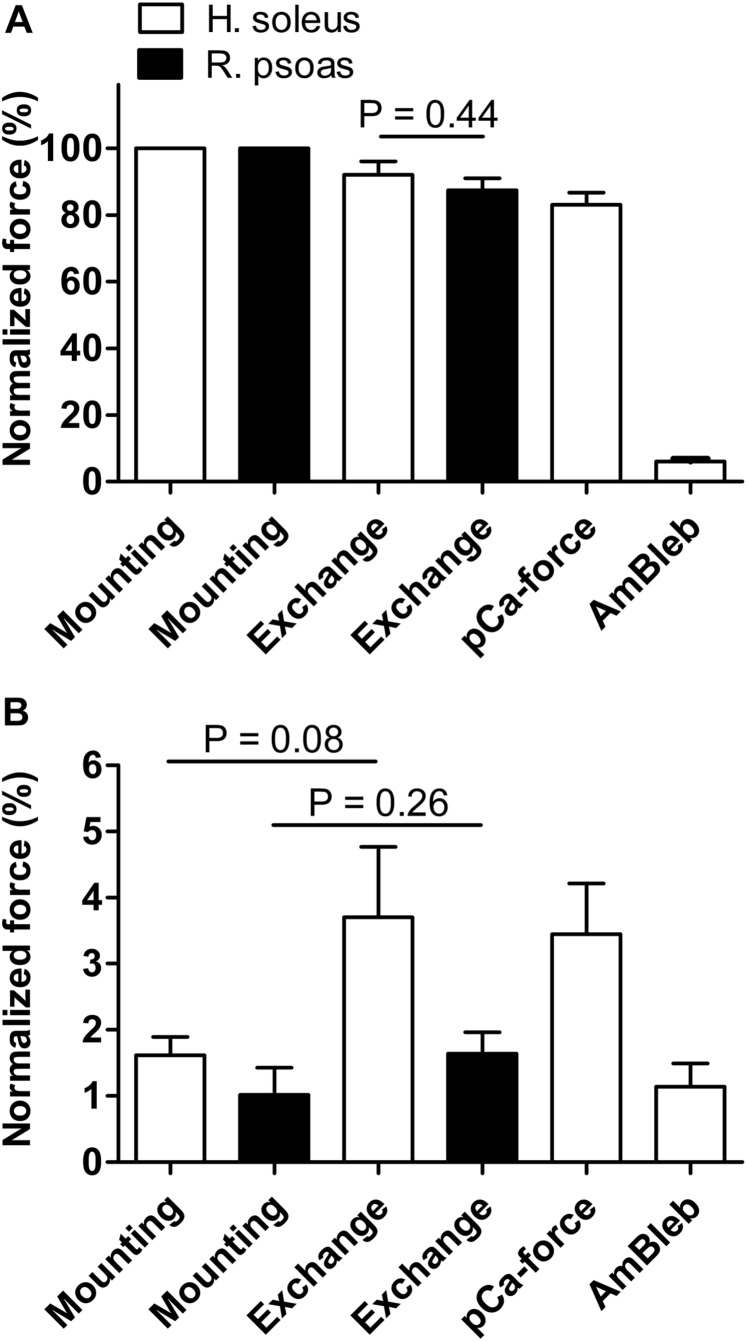
Effect of fsTn-IANBD exchange and of the experimental protocol on force generation in slow-twitch human soleus (*n* = 8) and fast-twitch rabbit psoas (*n* = 5) muscle fibers. **(A)** Normalized maximal force (pCa 4.5) after mounting the fiber, after troponin exchange, after measuring the pCa-force relationship (before AmBleb experiments), and after incubation in 50 μM AmBleb. 100% corresponds to maximal force before fsTn-IANBD exchange. Note that maximal force after troponin exchange is not significantly different between both preparations. **(B)** Same as in **A** for force at pCa 7.5. Note that increases in force are not statistically significant.

Isometric force generation and *k*_tr_ were quantified immediately after mounting the fibers, after the troponin exchange procedure, and after completing the first part of the experiment (without AmBleb). Maximum force (pCa 4.5) was slightly reduced by troponin exchange and thereafter (Figure 2A). Resting force (pCa 7.5) was slightly increased by troponin exchange (Figure 2B). A similar reduction in maximal force and increase of resting force also occurred with the troponin exchange procedure in fast-twitch rabbit fibers (Figures 2A,B), suggesting that these effects result from the exchange procedure itself rather than from the exchange of the fsTn-IANBD in a different muscle type, i.e., of the rabbit fast-twitch troponin in human slow-twitch muscle.

**FIGURE 3:**
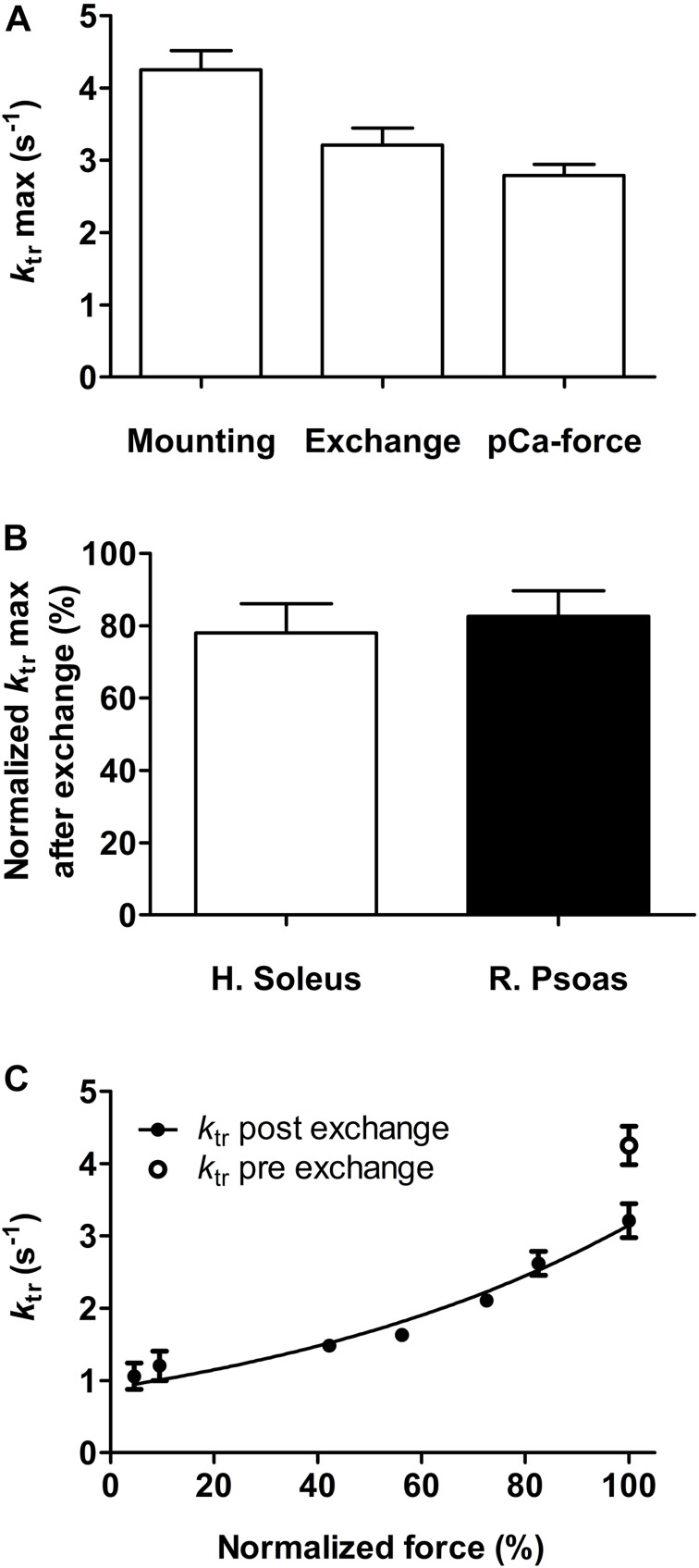
Effect of fsTn-IANBD exchange and of the experimental protocol on the rate constant of force redevelopment, *k*_tr_. **(A)** *k*_tr_ max (pCa 4.5) after mounting the fiber, after troponin exchange, and after measuring the pCa-force relationship (before AmBleb experiments) in human soleus slow-twitch muscle fibers (*n* = 8). **(B)** Comparison of *k*_tr_ max (pCa 4.5) after fsTn-IANBD exchange in slow-twitch (human soleus) and fast-twitch (rabbit psoas, *n* = 5) fibers. 100% corresponds to maximal *k*_tr_ before fsTn-IANBD exchange. **(C)** Force–*k*_tr_ relationship after fsTn-IANBD exchange in slow-twitch fibers. Force is normalized to force at pCa 4.5. *k*_tr_ max before troponin exchange is shown for direct comparison.

After mounting the fiber, *k*_tr_ determined in activating solution was 4.25 ± 0.27 s^–1^ (*n* = 8). This value was reduced to 3.21 ± 0.23 s^–1^ (*n* = 8) after the troponin exchange, and to 2.79 ± 0.15 s^–1^ (*n* = 8) after completing the first part of the experiment (Figure 3A). Fast-twitch rabbit fibers exhibited the same diminution (Figure 3B). The reduced force and *k*_tr_ values observed here were within the usual range of rundown obtained in our previous work (Brenner et al., 1999) and in other reports (Ferguson et al., 2003; Sun et al., 2006). All exchanged fibers exhibited a rather flat force–*k*_tr_ relationship typical of slow-twitch fibers (Figure 3C) (Metzger and Moss, 1990; Gordon et al., 2000). Additionally, reported *k*_tr_ of fast-twitch rabbit psoas fibers at 15 and 25°C were ≈15 and ≈25 s^–1^, respectively (Brenner and Eisenberg, 1986). Thus, the *k*_tr_ of ≈4 and ≈3 s^–1^ reported here at 20°C before and after exchange and using the same methodology are clearly distinctive of slow-twitch fibers and are not affected by the troponin isoform. Mean and SEM values showed in Figures 2, 3 are presented in Supplementary Table S1.

### Changes in IANBD Emission During Loaded Isometric Contraction and Isotonic Contraction

**FIGURE 4:**
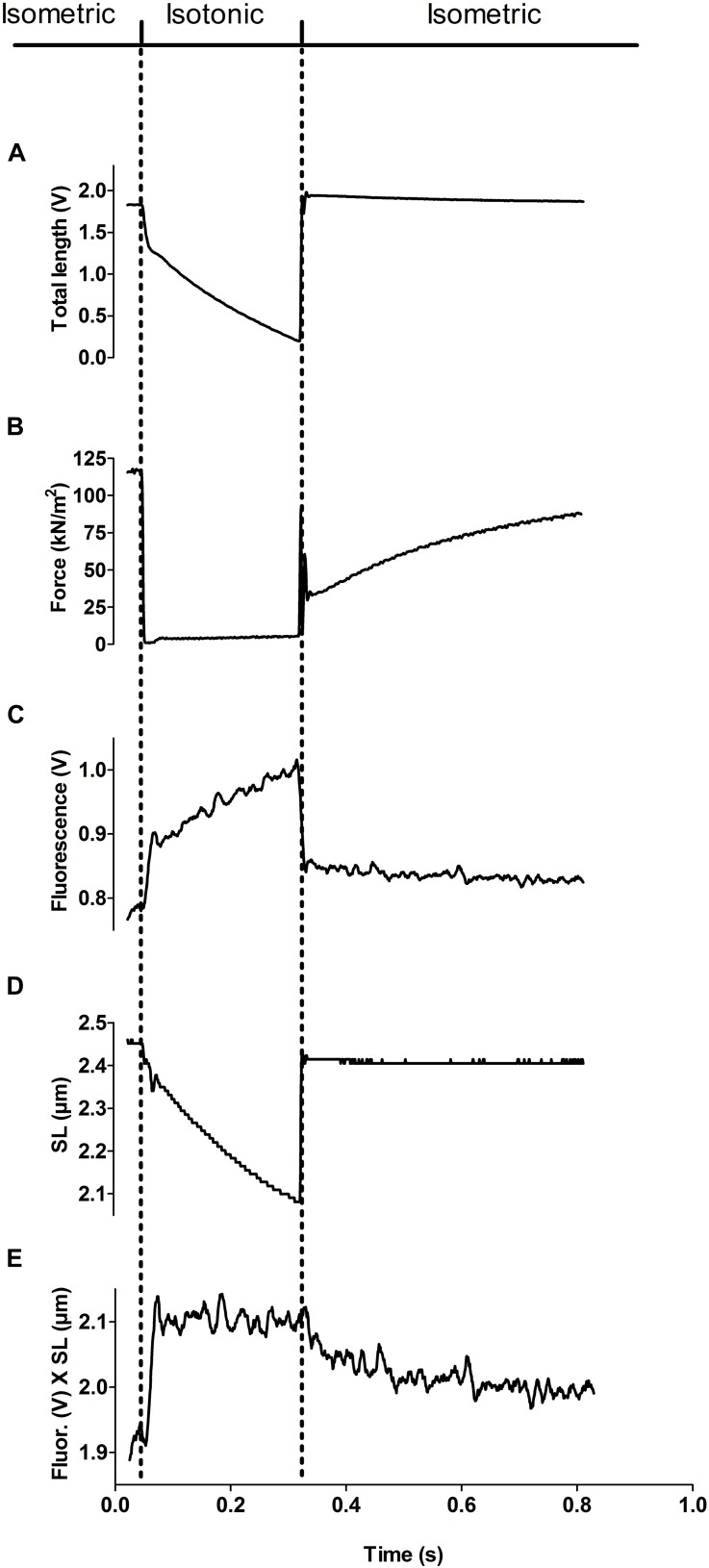
Experimental protocol for measuring steady state activation and dynamic inactivation of the thin filament. The dotted lines show the time points of the switch from isometric to isotonic (slightly loaded) and back to isometric conditions. **(A)** Total length of the muscle fiber (expressed in length transducer signal voltage). **(B)** Force signal. Note that the fiber does not slack during the isotonic phase, since a constant, small amount of tension is kept. **(C)** N-((2-(Iodoacetoxy)ethyl)-N-methyl)amino-7-nitrobenz-2-oxa-1,3-diazole (IANBD) emission intensity during isometric and isotonic phases. Note the difference in intensity immediately before and immediately after the isotonic phase. **(D)** Sarcomere length during isometric and isotonic phases was used as a correction factor for IANBD emission intensity. **(E)** IANBD emission intensity multiplied by sarcomere length (SL). This corrected IANBD emission does not show any step at the transitions between isotonic and isometric contraction. Thus, this signal shows only emission changes induced by inactivation (during isotonic phase) and reactivation (during the subsequent isometric phase) of the troponin complex while excludes alterations induced by changes in the SL (compared it with the non-corrected IANBD emission in **C**, especially in the transition from isotonic to isometric contraction). All traces in this figure represent averages of six added transients.

We applied a protocol to the fsTn-IANBD-exchanged slow-twitch fibers to successively measure at different calcium concentrations: (1) steady state force generation and steady state IANBD emission intensity during isometric contraction, (2) dynamic changes in IANBD emission intensity and force upon switching from isometric conditions (high load) to isotonic shortening at very low loads (≈5% of isometric force), and (3) the corresponding dynamic changes upon re-stretching the fiber to the original fiber length and filament overlap (Figure 4). Changes in fiber length and active force during such transitions are shown in Figures 4A,B, respectively. When switching from isometric contraction to isotonic shortening, a fast increase in the IANBD emission intensity was followed by a slow increase (Figure 4C). After correction of IANBD emission intensity for SL changes (see section “Materials and Methods”), whereby IANBD emission intensity is normalized to the amount of fsTn-IANBD in the excited fiber segment, only the fast increase at the beginning of the isotonic phase remained, and the slow increase disappeared (Figures 4D,E). This shows that the slow increase resulted solely from SL change and not from intrinsic changes in fsTn-IANBD fluorescence properties. The fast increase at the beginning of the isotonic phase was therefore interpreted to reflect the rapid deactivation kinetics of fsTn-IANBD induced by rapid redistribution of cross-bridges from force-generating to non-force-generating states, as previously shown for fast-twitch rabbit psoas fibers (Brenner and Chalovich, 1999). The increase in IANBD emission induced by mechanical unloading (MU) was reversed during the period of force redevelopment after switching back to isometric conditions. We interpret this, as in the past, as myosin-induced activation of the thin filament (Brenner and Chalovich, 1999). The observed rate constants *k*_obs_ of fluorescence decrease and *k*_tr_ during the isometric phase support this view since they are not significantly different from each other (*p* = 0.052): at pCa 4.5, mean *k*_tr_ and mean rate constant of fluorescence decrease are 3.21 ± 0.23 s^–1^ (*n* = 8) and 3.93 ± 0.24 s^–1^ (*n* = 8), respectively. These interpretations of the fluorescence signals during the isotonic and isometric phases are consistent with other reports (Fusi et al., 2014).

### Inhibition of the Activating Effect of Myosin on Thin Filament Activation by AmBleb

**FIGURE 5:**
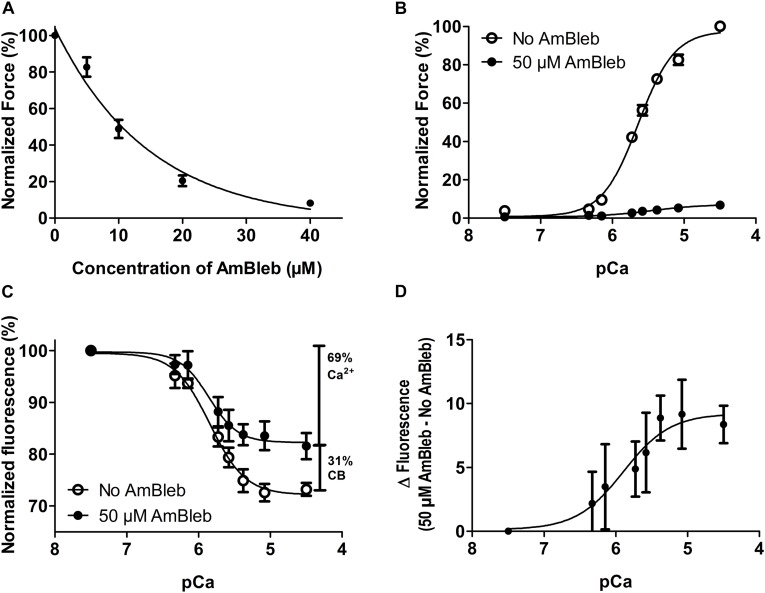
Steady state force and thin filament activation with and without the activating effect of myosin at different calcium concentrations in human slow-twitch fibers. **(A)** Inhibition of active force by incubation in increasing AmBleb concentration. *n* = 4. **(B)** pCa-force relationship in exchanged muscle fibers before (empty circles) and after (filled circles) incubation in 50 μM AmBleb. **(C)** pCa-IANBD emission intensity relationship before (empty circles) and after (filled circles) incubation in 50 μM AmBleb. Note the clearly diminished activation of the thin filament throughout the whole pCa scale when it is induced only by calcium (after incubation in AmBleb). The vertical bars show the relative magnitude of the thin filament activation induced by calcium and strong binding cross-bridges (CB). The combination of both factors (100%) accounts for the total fluorescence signal observed between pCa 7.5 and 4.5. **(D)** Subtracting the fluorescence signals in **C** reveals that AmBleb treatment increased the fluorescence emission intensity (less activation of the thin filament) and that the myosin contribution to thin filament activation increases with the calcium concentration. n = 8 in **B**, **C**, and **D**. All AmBleb incubations were made for 10 min in relaxing solution, before force and fluorescence measurements. For direct comparisons of force and thin filament activation, see Supplementary Figure S2.

Since we wanted to separate the effects of calcium activation from activating effects of force producing myosin binding to the thin filament, we used AmBleb to inhibit myosin heads from entering the strong binding, force-generating states. We determined in a separate group of muscle fibers, the AmBleb concentration required to effectively prevent the formation of the strong binding states, as determined by its inhibition of force development (Figure 5A). Knowing this concentration, we were able to perform the experiment depicted in Figure 4 also after incubating the exchanged fibers for 10 min in 50 μM AmBleb in relaxing solution.

### Effect of Myosin Heads on Steady State Thin Filament Activation at Different Calcium Concentrations

Calcium-dependent steady state force development and emission intensity of fsTn-IANBD measurements are shown in Figure 5 and Table 1. As expected, isometric force development of the exchanged muscle fibers was highly inhibited after incubation in relaxing solution with 50 μM AmBleb for 10 min (Figure 5B; note the strong and uniform force inhibition achieved in all fibers, as evidenced by the very small SEM bars). Increasing the calcium concentration diminished the IANBD emission intensity (Figure 5C in No-AmBleb condition and Table 1), which represents the activation of the thin filament as we previously reported for fast-twitch rabbit psoas fibers and myofibrils (Brenner and Chalovich, 1999; Lopez-Davila et al., 2012). Compared to the No-AmBleb condition the IANBD emission observed when increasing the calcium concentration was clearly increased after incubation with 50 μM AmBleb, meaning less thin filament activation in the transition from pCa 7.5 to 4.5. At pCa 4.5, this increase represents ≈31% of the total fluorescence change observed between relaxing and saturating calcium concentrations in the No-AmBleb condition (see the vertical bars in Figure 5C) and strongly suggests that in human slow-twitch soleus fibers, force-generating cross-bridges contribute to thin filament activation. Note that the contribution of force-generating cross-bridges to the thin filament activation increases with the calcium concentration (Figures 5C,D). Despite the tendency to reduce calcium sensitivity (pCa_50_) of force development, AmBleb treatment significantly modified neither this variable nor its cooperativity (n_H_). Similarly, AmBleb treatment modified neither calcium sensitivity nor cooperativity of thin filament activation as measured by fsTn-IANBD emission intensity (see Table 1).

**TABLE 1:** Steady state force and fluorescence changes.

Parameter	Force	Force 50	Fluorescence	Fluorescence 50
		μM AmBleb		μM AmBleb
pCa_50_	5.60 ± 0.02	5.48 ± 0.07	5.84 ± 0.08	5.90 ± 0.07
n_H_	1.77 ± 0.06	1.53 ± 0.26	1.85 ± 0.12	1.82 ± 0.40
Value at pCa 4.5*	100	6.77 ± 0.88†	73.2 ± 1.25	81.5 ± 2.51††

*Force values are normalized to force generated at pCa 4.5 after IANBD-labeled fsTn (fsTn-IANBD) exchange. Fluorescence values are normalized to emission intensity obtained at pCa 7.5 after fsTn-IANBD exchange. ^†^Significantly different to force before AmBleb incubation (*p* = 0.0001). ^††^Significantly different to fluorescence emission before AmBleb incubation (*p* = 0.0007). *n* = 8 fibers.

### Dynamic Inactivation of the Thin Filament by Lowering the Population of Strong Binding Myosin Heads at Different Calcium Concentrations

**FIGURE 6:**
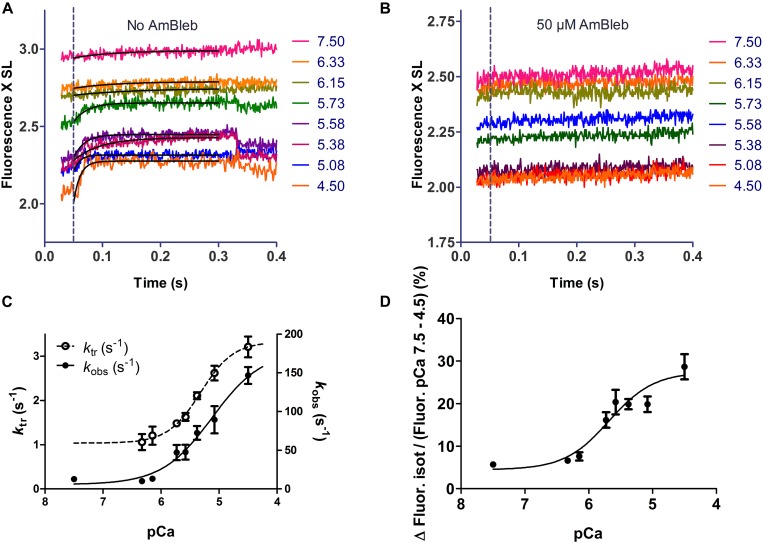
Unloading-induced deactivation of the thin filament. **(A)** Example of isotonic shortening phases of a single muscle fiber. Increasing the concentration of calcium induced a 29.6% decrease of fluorescence intensity (from 2.94 V at pCa 7.5 to 2.07 V at pCa 4.5). The dotted line shows the transition from isometric to isotonic contraction. **(B)** Same as in **A** after 10 min incubation in 50 μM AmBleb in relaxing solution. Increasing the calcium concentration induced only an 18.9% decrease of fluorescence intensity (from 2.48 V at pCa 7.5 to 2.01 V at pCa 4.5). **(C)** The rate constant of the thin filament deactivation induced by switching from isometric contraction to isotonic shortening phase [observed rate constant (*k*_obs_)] increased from ≈10 to ≈ 150 s^– 1^ between pCa 7.5 and 4.5. Note that the rate constant of force redevelopment after re-stretching the fiber back to isometric conditions (*k*_tr_) is ≈10–50-fold lower than *k*_obs_ at same calcium concentration. **(D)** The amplitude of increase in IANBD emission induced by unloading is normalized to the delta of emission intensity between pCa 7.5 and 4.5. All traces in **A** and **B** represent averages over of six cycles. In **C** and **D**, *n* = 8.

Dynamic changes of force development and emission intensity of fsTn-IANBD are presented in Figure 6 and Table 2. Upon switching from isometric contraction to isotonic shortening, the thin filament becomes partially deactivated as evidenced by the increase in the IANBD emission intensity (Figure 6A). Mean *k*_obs_ of this emission increase ranged between ≈15 and ≈150 s^–1^ at pCa 7.5 and 4.5, respectively (Figure 6C). The amplitude of the IANBD emission increase ranged between ≈5 and ≈29% of the total emission change observed between pCa 7.5 and 4.5 (Figure 6D and Table 2). Note that at saturating calcium, the magnitude of this fluorescence increase (indicating partial inactivation of the thin filament) is virtually the same obtained in steady state conditions by pretreatment with AmBleb (Figure 5C, vertical bars). *k*_tr_ values ranged between 1 and 3 s^–1^ between pCa 7.5 and 4.5, respectively (Figure 6C and Table 2). After AmBleb incubation, fluorescence emission decreased only in steady state with the increasing calcium concentration, since isotonic shortening-induced inactivation was almost completely abolished (Figure 6B). These results strongly suggest that strong binding states of cycling cross-bridges contribute to activation of the thin filament. Interestingly, the calcium sensitivity (pCa_50_) of the amplitude of the fluorescence increase, induced by switching from high load to low load (Table 2), is similar to the pCa_50_ of force (Table 1), whereas the pCa_50_ of total fluorescence and the fluorescence increase induced by AmBleb are higher (Table 1). These results indicate that the total IANBD fluorescence and the AmBleb-induced fluorescence increase but not the unloading-induced fluorescence increase include some changes in fsTn of high calcium sensitivity uncoupled from force regulation.

**TABLE 2:** Dynamic force and fluorescence changes.

Parameter	*k*_tr_ (Force	*k*_obs_ (Fluorescence	Amplitude
	redevelopment)	increase)	(fluorescence
			increase)
pCa_50_	5.37 ± 0.04	5.44 ± 0.01	5.69 ± 0.11
n_H_	1.75 ± 0.24	2.04 ± 0.73	1.72 ± 0.26
Value at pCa 4.5	3.21 ± 0.23	147 ± 11^†^	29 ± 2.9*

^†^Significantly different than *k*_tr_ (*p* < 0.0001). *Amplitude normalized to the total reduction of emission intensity between pCa 7.5 and 4.5. *n* = 8 fibers.

## Discussion

### Synthesis

To the best of our knowledge, we have explored for the first time the effect of cycling cross-bridges on thin filament activation in human slow-twitch muscle fibers. Our results suggest that during isometric contraction, myosin heads significantly contribute to the activation of the thin filament. The strong binding states of cycling cross-bridges induce conformational changes sensed by Cys 133 in fsTn-IANBD. This myosin-induced activation of the thin filament was detected by reducing the occupation of strong binding states of the cross-bridges with two independent approaches: (1) the myosin inhibitor AmBleb and (2) suddenly switching from isometric contraction to isotonic shortening at low loads. In contrast to previous reports using the same probe for fast-twitch rabbit psoas muscle fibers, here in slow-twitch soleus fibers, saturating calcium concentration alone did not fully activate the thin filament, and the amount of myosin-induced activation increased with the activation level.

### Cys 133 of fsTnI Provides a Native Labeling Site for Probing Thin Filament Activation

We introduced a fluorescent probe on Cys 133 of the exchanged fsTn-IANBD as we did in other muscle preparations (Brenner et al., 1999; Lopez-Davila et al., 2012). In fsTnI, Cys 133 is located on a putative hinge in the C-terminal domain of troponin I (C-TnI) that connects the switch region (C-TnI-SR) with the mobile domain (C-TnI-MD) (Vinogradova et al., 2005). Furthermore, Cys 133 is in close proximity to the second actin-Tm binding region in C-TnI-MD (Tripet et al., 1997). Changes in fluorescence of this probe reflect conformational changes related to the state of activation of actin-tropomyosin-troponin (Greene, 1986). This probe does not appear to sense tension directly as changes in fluorescence occur also in solution when force is not produced (Trybus and Taylor, 1980; Greene, 1986; Brenner et al., 1999). Based on its location as well as on experimental and modeling-based evidence, we suggested that labels at this residue are sensitive to both fast interaction between C-TnI-SR and the N-terminal regulatory domain of troponin C (N-TnC) triggered by calcium binding to N-TnC and subsequent slow dissociation of C-TnI from actin (Brenner and Chalovich, 1999; Brenner et al., 1999; Lopez-Davila et al., 2012). Such a well-characterized sensor for the slow-twitch troponin complex is not available.

### Comparison With Previous Studies on Cardiac Fibers

Incubating the fibers in AmBleb produced a 31% decrease in the fluorescence signal associated with regulatory conformational changes at maximal activation (Figure 5C and Table 1). That observation implies that cycling cross-bridges contribute to thin filament activation in human slow-twitch fibers. Using vanadate to inhibit strong binding of myosin heads in isometrically contracting cardiac trabeculae, Smith et al. (2009) attributed 15% of the signal for activation to strong actomyosin interactions of cycling cross-bridges. Another vanadate study showed that cycling cross-bridges are responsible for approximately 30% of the opening of the hydrophobic patch of N-TnC in isometrically contracting papillary fibers (Rieck et al., 2013). A subsequent, similar study suggested that up to 45% of the detected thin filament activation could be explained by the strong actomyosin interactions of cycling cross-bridges (Li et al., 2014). Similarly, blebbistatin inhibited thin filament activation sensed by the change in orientation of the IT-arm by about 35% at saturating calcium in cardiac trabeculae (Robertson et al., 2015; Kampourakis et al., 2016, 2018). Thus, the value of 31% of myosin contribution to thin filament activation that we report here by means of AmBleb in slow-twitch fibers scales in the midpoint of the values of 15–45% reported for isometric contracting cardiac preparations. Differences in the magnitude of activation could be explained by the different probes used in those reports.

### Comparison With Previous Studies on Fast Skeletal Muscle Fibers

The mechanical maneuver of suddenly switching from isometric contraction to isotonic shortening, i.e., MU, has been previously used in studies on fast-twitch skeletal muscle fibers to decrease the population of strong binding states of cycling cross-bridges and their contribution to thin filament activation (Brenner and Chalovich, 1999; Fusi et al., 2014). In those studies, and in our case, this maneuver induced partial inactivation of the thin filament. By using the same probe as used here (fsTn-IANBD), Brenner and Chalovich reported that MU at sub-saturating calcium concentrations changed the fluorescence emission toward inactivation by 20% of the total fluorescence change observed between relaxing and saturating calcium concentrations. Only 3% change occurred at saturating calcium (Brenner and Chalovich, 1999). By using a bifunctional rhodamine probe attached to the IT arm of fsTn in fast-twitch rabbit fibers, Fusi et al. (2014) reported 15% inactivation by MU at saturating calcium. Thus, our value of 29% thin filament inactivation at saturating calcium reported here is higher than in those previous reports on fast-twitch fibers, especially when using the same probe. On the other hand, when the myosin inhibitor BTS was applied to isometrically contracting fast-twitch fibers, Fusi et al. reported 26% inactivation, only slightly below the 31% inactivation induced here by AmBleb in isometric conditions.

Altogether, it seems to apply that in human slow-twitch muscle fibers, the magnitude of myosin-induced thin filament activation detected by means of myosin inhibitors is similar to that previously reported for fast-twitch and cardiac animal tissues. In contrast, decreasing the occupation of myosin strong binding states by MU at saturating calcium induces a higher degree of inactivation of the thin filament in human slow-twitch than in rabbit fast-twitch fibers, especially when the same probe was used (Brenner and Chalovich, 1999; Fusi et al., 2014). These differences could be attributable to (a) the cardiac myosin head isoform present in slow-twitch human muscle fibers (Schiaffino and Reggiani, 2011), (b) proteins of the thin filament other than fsTn, since it is a common factor in Brenner and Chalovich’s (1999) report, Fusi et al.’s (2014) report, and in our present report, and (c) temperature, since it was 5°C in the publication of Brenner and Chalovich (3% inactivation at saturating calcium) and 12°C in the 2014 publication of Fusi et al. (15% inactivation).

Finally, we noted that the somewhat smaller amplitude of fluorescence change induced by MU (29%), in comparison with chemical inhibition (31%), is also observable in the report of Fusi et al. (2014) (15% by MU vs 26% by BTS). These small differences likely underlie the experimental approaches, which include long-term, permanent reduction of strongly bound myosin states under negligible cross-bridge cycling in the presence of inhibitors (AmBleb or BTS) and short-term, intermittent reduction of these states under ongoing cross-bridge cycling during MU. In this respect, Linari et al. (2015) reported that a short period of zero force shortening is not enough to fully reach the OFF structure of the thick filament (Linari et al., 2015), which might also prevent the full inactivation of the thin filament.

### Possible Steps Regulated by the Myosin Head

In our study, *k*_obs_ of thin filament inactivation induced by MU increased from ≈10 to ≈150 s^–1^ with increasing calcium concentrations. These values are close to those obtained for the *k*_obs_ of the slow phase of the biphasic fluorescence change observed in rabbit psoas myofibrils containing the same probe by rapid mixing with calcium containing buffers (Lopez-Davila et al., 2012). In the former study, a fast change with ≈10-fold higher rate constants was also observed. While the fast change was attributed to a fast interaction of C-TnI-SR with N-TnC triggered by calcium binding on troponin C, the slow phase was attributed to the dissociation of C-TnI from actin and tropomyosin. The similar kinetics of the fluorescence changes detected in the present study, in the previous study on fast-twitch fibers (Brenner and Chalovich, 1999), and in the slow phase in rabbit psoas myofibrils (Lopez-Davila et al., 2012) strongly suggest that the kinetics observed after MU primarily probes the dissociation of C-TnI from actin. This view is supported by other studies suggesting that the transition from weakly to strongly bound actomyosin states induces azimuthal movement of tropomyosin on actin, which in turn destabilizes the interaction between C-TnI and actin and stabilizes the thin filament activated state and vice versa (Rieck et al., 2013; Li et al., 2014).

Using labels on the IT arm, it has also been suggested that the activation is generated through a direct effect of the myosin head on the troponin complex (Kobayashi and Solaro, 2005; Fusi et al., 2014) or that the myosin head-induced azimuthal displacement of tropomyosin on actin in turn influences the TnT portion of the IT arm (Fusi et al., 2014). Our data are not in conflict with these possibilities. Indeed, the troponin I component of the IT arm is immediately upstream from the inhibitory region of C-TnI (Takeda et al., 2003) and thus very close to Cys 133. It has been additionally suggested that one of the main roles of the IT arm is to stabilize key interactions of C-TnI on the thin filament in skeletal muscle (Knowles et al., 2012) and probably in cardiac muscle since it is highly conserved among both muscle types (Takeda et al., 2003). All of these features of the IT arm could mean that both the IT and our Cys 133 labels sense highly related events of the myosin-induced activating effects, although from different locations inside or close to the IT arm, respectively.

### Effect of Replacing the Endogenous ssTn by fsTn-IANBD

The nature of the troponin complex defines the calcium sensitivity of force development. The introduction of probes on TnC of cardiac (Sun et al., 2009) and fast-twitch skeletal muscle preparations (Sun et al., 2006) have been shown to significantly decrease pCa_50_ of force development by up to 0.4 pCa units. However, since other key parameters of muscle contraction are conserved in those preparations, they are still highly representative of their respective muscle type and have been successfully used to study thin filament activation. We showed previously that labeling fsTn with IANBD neither modified pCa_50_ nor n_H_ of isometric force development, nor kinetic parameters of force development and relaxation (Lopez-Davila et al., 2012). On the other hand, exchanging the troponin isoform (e.g., fsTn by cardiac or ssTn isoforms) increases pCa_50_ of force development (Piroddi et al., 2003; de Tombe et al., 2007). Again, such troponin isoform exchange approaches did not modify other key parameters like maximum force, kinetics of the force rise (*k*_tr_), nor those of force decay following calcium removal (Piroddi et al., 2003; de Tombe et al., 2007; Stehle et al., 2007). Consistently with these models of troponin isoform exchange and insertion of probes, we showed in our controls that *k*_tr_ at saturating calcium, *k*_tr_-force curve, as well as maximal and resting force, are not modified by substitution of ssTn by fsTn-IANBD (Figures 2, 3). As such, our preparation is highly representative of slow-twitch muscle, even though we consider a troponin isoform-induced modification of the original pCa_50_ of force development very likely in our model.

When switching from isometric contraction to isotonic shortening, the rate constant of the conformational change of the fsTn troponin probe (*k*_obs_) is much faster than force redevelopment (*k*_tr_), which fits with previous reports using the same (Brenner and Chalovich, 1999; Lopez-Davila et al., 2012) and other probes (Fusi et al., 2014) in fast-twitch skeletal and cardiac muscle (Bell et al., 2006; Solzin et al., 2007). Though the kinetics of the native ssTn complex are unknown from our experiments and might be different from that of the fsTn probe, we note that exchange of this complex by the fsTn complex did not accelerate the rate of force redevelopment. Thus, our data contribute to complete the view that the thin filament activation is not the rate-limiting step in force development in all kinds of striated muscle, including human preparations.

Treatment with myosin inhibitors that stabilize the ADP. P_*i*_ state of myosin resulted in a decreased pCa_50_ of both force development (Martyn et al., 1999; Smith et al., 2009; Brandt and Poggesi, 2014) and thin filament activation, as measured by probes on troponin of cardiac and fast-twitch skeletal muscle (Sun et al., 2006; Rieck et al., 2013). The conserved pCa_50_ for those variables reported here after treatment with AmBleb could be a feature of (a) the slow-twitch fibers used, (b) the human tissue, (c) the nature of the hybrid system in our experiments, (d) the fsTn-IANBD probe, and (e) a combination of all or some of these. These possibilities should be the subject of detailed, systematic analysis in the future since they have important implications for regulation of muscle contraction. It is especially interesting that in our hands, even after exchanging rabbit fsTn for a human fsTn (wild type, without probes), in rabbit psoas fast-twitch fibers, we see a significantly decreased pCa_50_ of force development after incubation in 50 μM AmBleb (unpublished results). This opens the possibility that the conserved pCa_50_ value of force development after AmBleb treatment reported here could be indeed a feature of slow-twitch muscle. To explore this question, pCa–force relationships should be recorded at several AmBleb concentrations. This procedure would allow analyzing the changes of the pCa_50_ values of force development at progressive levels of force inhibition, since in our experiment the effect could be masked by the profound force inhibition that was achieved.

### Critical View of the Data

Our hybrid system incorporating an fsTn into a slow-twitch skeletal muscle fiber might not completely resemble the activation and inactivation kinetics of the native ssTn troponin complex in slow-twitch muscle. However, the well-known characterization of the steady state and dynamic behavior, including the previously shown sensitivity of the label to the myosin activating effect of fsTn-IANBD incorporated in fast-twitch skeletal muscle (Brenner and Chalovich, 1999; Brenner et al., 1999), makes it a good starting point to systematically compare regulation of muscle contraction among different muscle types. In this specific case, we used it as a sensor of a possible activating effect of the human myosin isoform present in slow-twitch skeletal and cardiac muscle.

The chemical and mechanical approaches used here to reduce the occupation of the strong binding states of the myosin head have been shown to trigger changes in the thick filament itself, such that the observed thin filament inactivation induced by those protocols could be explained by alternative mechanisms rather than by a direct effect of strong binding myosin on actin and tropomyosin. These facts, however, do not exclude the interpretation that actin bound myosin heads contribute to activation of the thin filament during steady state calcium activation, which is considered a fundamental property of thin filament regulation. For a comprehensive review, see Irving (2017).

### Summary and Outlook

Here we show by two different approaches, that at submaximal and maximal calcium concentrations, strong binding states of cycling cross-bridges significantly contribute to thin filament activation in human slow-twitch muscle fibers. To the best of our knowledge, this is the first study exploring this issue in slow-twitch skeletal muscle, which completes the view that myosin-induced activation is a conserved phenomenon in striated muscle, including human tissue.

Human cardiac myosin mutations are conserved in slow-twitch fibers of the human soleus muscle and are accessible in human patients (Kohler et al., 2002; Kirschner et al., 2005). Applying this protocol to such human soleus muscle biopsies will extend the understanding of molecular mechanisms triggered by point mutations in the sarcomere and finally leading to cardiomyopathies. Consistent with this model, it has been recently shown that the hypertrophic cardiomyopathy mutation R403Q of the beta myosin head induced the same kind of alterations in isometrically contracting myofibrils isolated both from ventricle and slow-twitch fibers of a rabbit model (Lowey et al., 2018). Similarly, our own studies on slow skeletal muscle fibers and cardiomyocytes from patients with β-cardiac myosin mutation R723G revealed a very similar reduction in calcium sensitivity in both preparations compared to controls (Kirschner et al., 2005; Kraft et al., 2013). How myosin mutations as well as small molecules affect thin filament activation in human muscle tissue and how this correlates with mechanical function will be subject of further research.

## Data Availability Statement

The raw data supporting the conclusions of this article will be made available by the authors, without undue reservation, to any qualified researcher.

## Ethics Statement

The studies involving human participants were reviewed and approved by the Hannover Medical School Ethics Committee (Vote No. 2729/2001). The patients/participants provided their written informed consent to participate in this study.

## Author Contributions

This project was the original idea of BB. He created the original concept (including SL correction of fluorescence transients), designed the experiment, and established the experimental setup. AL-D contributed to establish the experiment design, performed mechanical and fluorescence experiments, analyzed the data, and wrote the manuscript. JC created the original concept, contributed to data interpretation, and edited the manuscript. RS contributed to data interpretation, supervised the project, and contributed to writing the manuscript. SZ isolated and labeled fsTn-IANBD and contributed to Western blot experiments. BP performed Western blot experiments. FM isolated single slow-twitch human fibers from soleus muscle biopsies. AM-C and AR developed AmBleb, contributed to data interpretation, and edited the manuscript. TK supervised the project and edited the manuscript.

## Conflict of Interest

AM-C is the owner of Optopharma Ltd. and AR is part-time employed by Optopharma Ltd. Related patents: PCT/EP2017/051829, WO2017129782. The remaining authors declare that the research was conducted in the absence of any commercial or financial relationships that could be construed as a potential conflict of interest.

## Abbreviations

AmBleb: para-aminoblebbistatin
C-TnI: C-terminal domain of troponin I
C-TnI -MD: mobile domain of C-TnI
C-TnI -SR: switch region of C-TnI
fsTn: fast-twitch skeletal troponin complex
fsTnI: fast-twitch skeletal troponin I subunit
fsTn-IANBD: IANBD-labeled fsTn
IANBD: N-((2-(Iodoacetoxy)ethyl)-N-methyl)amino-7-nitrobenz-2-oxa-1,3-diazole
*k*_obs_: observed rate constant (here in fluorescence transients)
*k*_tr_: rate constant of isometric force redevelopment after a short period of isotonic shortening
MU: mechanical unloading
N-TnC: N-terminal regulatory domain of troponin C
SL: sarcomere length
ssTn: slow-twitch skeletal troponin complex
ssTnI: slow-twitch skeletal troponin I subunit
TnI: troponin I subunit.

## References

[B1] Baxley T. Johnson D. Pinto J. R. Chalovich J. M. (2017). Troponin C mutations partially stabilize the active state of regulated actin and fully stabilize the active state when paired with delta14 TnT. *Biochemistry* 56 2928–2937. 10.1021/acs.biochem.6b01092 28530094 PMC6448410

[B2] Bell M. G. Lankford E. B. Gonye G. E. Ellis-Davies G. C. Martyn D. A. Regnier M. (2006). Kinetics of cardiac thin-filament activation probed by fluorescence polarization of rhodamine-labeled troponin C in skinned guinea pig trabeculae. *Biophys. J.* 90 531–543. 10.1529/biophysj.105.072769 16258047 PMC1367058

[B3] Brandt P. W. Poggesi C. (2014). Clusters of bound Ca(2+) initiate contraction in fast skeletal muscle. *Arch. Biochem. Biophys.* 552–553 60–67. 10.1016/j.abb.2013.12.013 24374032

[B4] Bremel R. D. Murray J. M. Weber A. (1973). Manifestations of cooperative behavior in regulated actin filament during actin-activated atp hydrolysis in presence of calcium. *Cold Spring Harb. Symp. Quant. Biol.* 37 267–274.

[B5] Brenner B. (1983). Technique for stabilizing the striation pattern in maximally calcium-activated skinned rabbit psoas fibers. *Biophys. J.* 41 99–102. 10.1016/s0006-3495(83)84411-7 6824759 PMC1329019

[B6] Brenner B. Chalovich J. M. (1999). Kinetics of thin filament activation probed by fluorescence of N-((2-(iodoacetoxy)ethyl)-N-methyl)amino-7-nitrobenz-2-oxa-1,3-diazole-labeled troponin I incorporated into skinned fibers of rabbit psoas muscle: implications for regulation of muscle contraction. *Biophys. J.* 77 2692–2708. 10.1016/s0006-3495(99)77103-1 10545369 PMC1201417

[B7] Brenner B. Eisenberg E. (1986). Rate of force generation in muscle: correlation with actomyosin ATPase activity in solution. *Proc. Natl. Acad. Sci. U.S.A.* 83 3542–3546. 10.1073/pnas.83.10.3542 2939452 PMC323553

[B8] Brenner B. Kraft T. Yu L. C. Chalovich J. M. (1999). Thin filament activation probed by fluorescence of N-((2-(iodoacetoxy)ethyl)-N-methyl)amino-7-nitrobenz-2-oxa-1,3-diazole-labeled troponin I incorporated into skinned fibers of rabbit psoas muscle. *Biophys. J.* 77 2677–2691. 10.1016/s0006-3495(99)77102-x 10545368 PMC1300542

[B9] Chalovich J. M. Greene L. E. Eisenberg E. (1983). Crosslinked myosin subfragment 1: a stable analogue of the subfragment-1.ATP complex. *Proc. Natl. Acad. Sci. U.S.A.* 80 4909–4913. 10.1073/pnas.80.16.4909 6576363 PMC384156

[B10] de Tombe P. P. Belus A. Piroddi N. Scellini B. Walker J. S. Martin A. F. (2007). Myofilament calcium sensitivity does not affect cross-bridge activation-relaxation kinetics. *Am. J. Physiol. Regul. Integr. Comp. Physiol.* 292 R1129–R1136. 17082350 10.1152/ajpregu.00630.2006

[B11] Eisenberg E. Weihing R. R. (1970). Effect of skeletal muscle native tropomyosin on the interaction of amoeba actin with heavy meromyosin. *Nature* 228 1092–1093. 10.1038/2281092a0 4249428

[B12] Ferguson R. E. Sun Y. B. Mercier P. Brack A. S. Sykes B. D. Corrie J. E. (2003). In situ orientations of protein domains: troponin C in skeletal muscle fibers. *Mol. Cell* 11 865–874. 12718873 10.1016/s1097-2765(03)00096-0

[B13] Fusi L. Brunello E. Sevrieva I. R. Sun Y. B. Irving M. (2014). Structural dynamics of troponin during activation of skeletal muscle. *Proc. Natl. Acad. Sci. U.S.A.* 111 4626–4631. 10.1073/pnas.1321868111 24616505 PMC3970506

[B14] Gordon A. M. Homsher E. Regnier M. (2000). Regulation of contraction in striated muscle. *Physiol. Rev.* 80 853–924. 10747208 10.1152/physrev.2000.80.2.853

[B15] Greene L. (1986). Cooperative binding of myosin subfragment one to regulated actin as measured by fluorescence changes of troponin I modified with different fluorophores. *J. Biol. Chem.* 261 1279–1285. 3753701

[B16] Hill T. L. Eisenberg E. Chalovich J. M. (1981). Theoretical models for cooperative steady-state ATPase activity of myosin subfragment-1 on regulated actin. *Biophys. J.* 35 99–112. 10.1016/s0006-3495(81)84777-7 6455170 PMC1327506

[B17] Houmeida A. Heeley D. H. Belknap B. White H. D. (2010). Mechanism of regulation of native cardiac muscle thin filaments by rigor cardiac myosin-S1 and calcium. *J. Biol. Chem.* 285 32760–32769. 10.1074/jbc.M109.098228 20696756 PMC2963418

[B18] Irving M. (2017). Regulation of contraction by the thick filaments in skeletal muscle. *Biophys. J.* 113 2579–2594. 10.1016/j.bpj.2017.09.037 29262355 PMC5770512

[B19] Johnson D. Angus C. W. Chalovich J. M. (2018). Stepwise C-terminal truncation of cardiac troponin T alters function at low and saturating Ca(2). *Biophys. J.* 115 702–712. 10.1016/j.bpj.2018.06.028 30057009 PMC6104287

[B20] Kampourakis T. Sun Y. B. Irving M. (2016). Myosin light chain phosphorylation enhances contraction of heart muscle via structural changes in both thick and thin filaments. *Proc. Natl. Acad. Sci. U.S.A.* 113 E3039–E3047. 10.1073/pnas.1602776113 27162358 PMC4889392

[B21] Kampourakis T. Zhang X. Sun Y. B. Irving M. (2018). Omecamtiv mercabil and blebbistatin modulate cardiac contractility by perturbing the regulatory state of the myosin filament. *J. Physiol.* 596 31–46. 10.1113/JP275050 29052230 PMC5746517

[B22] Kirschner S. E. Becker E. Antognozzi M. Kubis H. P. Francino A. Navarro-Lopez F. (2005). Hypertrophic cardiomyopathy-related beta-myosin mutations cause highly variable calcium sensitivity with functional imbalances among individual muscle cells. *Am. J. Physiol. Heart Circ. Physiol.* 288 H1242–H1251. 15550524 10.1152/ajpheart.00686.2004

[B23] Knowles A. C. Irving M. Sun Y. B. (2012). Conformation of the troponin core complex in the thin filaments of skeletal muscle during relaxation and active contraction. *J. Mol. Biol.* 421 125–137. 10.1016/j.jmb.2012.05.005 22579625

[B24] Kobayashi T. Solaro R. J. (2005). Calcium, thin filaments, and the integrative biology of cardiac contractility. *Annu. Rev. Physiol.* 67 39–67. 10.1146/annurev.physiol.67.040403.114025 15709952

[B25] Kohler J. Winkler G. Schulte I. Scholz T. McKenna W. Brenner B. (2002). Mutation of the myosin converter domain alters cross-bridge elasticity. *Proc. Natl. Acad. Sci. U.S.A.* 99 3557–3562. 10.1073/pnas.062415899 11904418 PMC122562

[B26] Kraft T. Chalovich J. M. Yu L. C. Brenner B. (1995). Parallel inhibition of active force and relaxed fiber stiffness by caldesmon fragments at physiological ionic strength and temperature conditions: additional evidence that weak cross-bridge binding to actin is an essential intermediate for force generation. *Biophys. J.* 68 2404–2418. 10.1016/s0006-3495(95)80423-6 7647245 PMC1282151

[B27] Kraft T. Witjas-Paalberends E. R. Boontje N. M. Tripathi S. Brandis A. Montag J. (2013). Familial hypertrophic cardiomyopathy: functional effects of myosin mutation R723G in cardiomyocytes. *JMCC* 57 13–22. 10.1016/j.yjmcc.2013.01.001 23318932

[B28] Li K. L. Rieck D. Solaro R. J. Dong W. (2014). In situ time-resolved FRET reveals effects of sarcomere length on cardiac thin-filament activation. *Biophys. J.* 107 682–693. 10.1016/j.bpj.2014.05.044 25099807 PMC4129473

[B29] Linari M. Brunello E. Reconditi M. Fusi L. Caremani M. Narayanan T. (2015). Force generation by skeletal muscle is controlled by mechanosensing in myosin filaments. *Nature* 528 276–279. 10.1038/nature15727 26560032

[B30] Lopez-Davila A. J. Elhamine F. Ruess D. F. Papadopoulos S. Iorga B. Kulozik F. P. (2012). Kinetic mechanism of Ca(2)(+)-controlled changes of skeletal troponin I in psoas myofibrils. *Biophys. J.* 103 1254–1264. 10.1016/j.bpj.2012.08.022 22995498 PMC3446660

[B31] Lowey S. Bretton V. Joel P. B. Trybus K. M. Gulick J. Robbins J. (2018). Hypertrophic cardiomyopathy R403Q mutation in rabbit beta-myosin reduces contractile function at the molecular and myofibrillar levels. *Proc. Natl. Acad. Sci. U.S.A.* 115 11238–11243. 10.1073/pnas.1802967115 30322937 PMC6217429

[B32] Martyn D. A. Freitag C. J. Chase P. B. Gordon A. M. (1999). Ca2+ and cross-bridge-induced changes in troponin C in skinned skeletal muscle fibers: effects of force inhibition. *Biophys. J.* 76 1480–1493. 10.1016/s0006-3495(99)77308-x 10049329 PMC1300125

[B33] McKillop D. F. Geeves M. A. (1993). Regulation of the interaction between actin and myosin subfragment 1: evidence for three states of the thin filament. *Biophys. J.* 65 693–701. 10.1016/s0006-3495(93)81110-x 8218897 PMC1225772

[B34] Metzger J. M. Moss R. L. (1990). Calcium-sensitive cross-bridge transitions in mammalian fast and slow skeletal muscle fibers. *Science* 247 1088–1090. 10.1126/science.2309121 2309121

[B35] Neulen A. Stehle R. Pfitzer G. (2009). The cardiac troponin C mutation Leu29Gln found in a patient with hypertrophic cardiomyopathy does not alter contractile parameters in skinned murine myocardium. *Basic Res. Cardiol.* 104 751–760. 10.1007/s00395-009-0038-y 19506933 PMC2758205

[B36] Piroddi N. Tesi C. Pellegrino M. A. Tobacman L. S. Homsher E. Poggesi C. (2003). Contractile effects of the exchange of cardiac troponin for fast skeletal troponin in rabbit psoas single myofibrils. *J. Physiol.* 552(Pt 3) 917–931. 10.1113/jphysiol.2003.051615 12937281 PMC2343446

[B37] Rauscher A. A. Gyimesi M. Kovacs M. Malnasi-Csizmadia A. (2018). Targeting myosin by blebbistatin derivatives: optimization and pharmacological potential. *Trends Biochem. Sci.* 43 700–713. 10.1016/j.tibs.2018.06.006 30057142

[B38] Rieck D. C. Li K. L. Ouyang Y. Solaro R. J. Dong W. J. (2013). Structural basis for the in situ Ca(2+) sensitization of cardiac troponin C by positive feedback from force-generating myosin cross-bridges. *Arch. Biochem. Biophys.* 537 198–209. 10.1016/j.abb.2013.07.013 23896515 PMC3836555

[B39] Robertson I. M. Sevrieva I. Li M. X. Irving M. Sun Y. B. Sykes B. D. (2015). The structural and functional effects of the familial hypertrophic cardiomyopathy-linked cardiac troponin C mutation, L29Q. *J. Mol. Cell. Cardiol.* 87 257–269. 10.1016/j.yjmcc.2015.08.017 26341255 PMC4640586

[B40] Schiaffino S. Reggiani C. (2011). Fiber types in mammalian skeletal muscles. *Physiol. Rev.* 91 1447–1531. 10.1152/physrev.00031.2010 22013216

[B41] Seebohm B. Matinmehr F. Kohler J. Francino A. Navarro-Lopez F. Perrot A. (2009). Cardiomyopathy mutations reveal variable region of myosin converter as major element of cross-bridge compliance. *Biophys. J.* 97 806–824. 10.1016/j.bpj.2009.05.023 19651039 PMC2718155

[B42] Shadrin I. Y. Khodabukus A. Bursac N. (2016). Striated muscle function, regeneration, and repair. *Cell. Mol. Life Sci.* 73 4175–4202. 10.1007/s00018-016-2285-z 27271751 PMC5056123

[B43] She M. Trimble D. Yu L. C. Chalovich J. M. (2000). Factors contributing to troponin exchange in myofibrils and in solution. *J. Muscle Res. Cell Motil.* 21 737–745. 11392555 10.1023/a:1010300802980

[B44] Sheng J. J. Jin J. P. (2014). Gene regulation, alternative splicing, and posttranslational modification of troponin subunits in cardiac development and adaptation: a focused review. *Front. Physiol.* 5:165. 10.3389/fphys.2014.00165 24817852 PMC4012202

[B45] Smith L. Tainter C. Regnier M. Martyn D. A. (2009). Cooperative cross-bridge activation of thin filaments contributes to the Frank-Starling mechanism in cardiac muscle. *Biophys. J.* 96 3692–3702. 10.1016/j.bpj.2009.02.018 19413974 PMC3325146

[B46] Solzin J. Iorga B. Sierakowski E. Gomez Alcazar D. P. Ruess D. F. Kubacki T. (2007). Kinetic mechanism of the Ca2+-dependent switch-on and switch-off of cardiac troponin in myofibrils. *Biophys. J.* 93 3917–3931. 10.1529/biophysj.107.111146 17704185 PMC2099212

[B47] Stehle R. Iorga B. Pfitzer G. (2007). Calcium regulation of troponin and its role in the dynamics of contraction and relaxation. *Am. J. Physiol. Regul. Integr. Comp. Physiol.* 292 R1125–R1128. 17158261 10.1152/ajpregu.00841.2006

[B48] Sun Y. B. Brandmeier B. Irving M. (2006). Structural changes in troponin in response to Ca2+ and myosin binding to thin filaments during activation of skeletal muscle. *Proc. Natl. Acad. Sci. U.S.A.* 103 17771–17776. 10.1073/pnas.0605430103 17101992 PMC1693822

[B49] Sun Y. B. Lou F. Irving M. (2009). Calcium- and myosin-dependent changes in troponin structure during activation of heart muscle. *J. Physiol.* 587 155–163. 10.1113/jphysiol.2008.164707 19015190 PMC2670030

[B50] Takeda S. Yamashita A. Maeda K. Maeda Y. (2003). Structure of the core domain of human cardiac troponin in the Ca(2+)-saturated form. *Nature* 424 35–41. 10.1038/nature01780 12840750

[B51] Tripet B. Van Eyk J. E. Hodges R. S. (1997). Mapping of a second actin-tropomyosin and a second troponin C binding site within the C terminus of troponin I, and their importance in the Ca2+-dependent regulation of muscle contraction. *J. Mol. Biol.* 271 728–750. 10.1006/jmbi.1997.1200 9299323

[B52] Trybus K. M. Taylor E. W. (1980). Kinetic studies of the cooperative binding of subfragment 1 to regulated actin. *Proc. Natl. Acad. Sci. U.S.A.* 77 7209–7213. 10.1073/pnas.77.12.7209 6938966 PMC350471

[B53] Varkuti B. H. Kepiro M. Horvath I. A. Vegner L. Rati S. Zsigmond A. (2016). A highly soluble, non-phototoxic, non-fluorescent blebbistatin derivative. *Sci. Rep.* 6:26141. 10.1038/srep26141 27241904 PMC4886532

[B54] Vinogradova M. V. Stone D. B. Malanina G. G. Karatzaferi C. Cooke R. Mendelson R. A. (2005). Ca(2+)-regulated structural changes in troponin. *Proc. Natl. Acad. Sci. U.S.A.* 102 5038–5043. 10.1073/pnas.0408882102 15784741 PMC555973

[B55] Wijnker P. J. Sequeira V. Foster D. B. Li Y. Dos Remedios C. G. Murphy A. M. (2014). Length-dependent activation is modulated by cardiac troponin I bisphosphorylation at Ser23 and Ser24 but not by Thr143 phosphorylation. *Am. J. Physiol. Heart Circ. Physiol.* 306 H1171–H1181. 10.1152/ajpheart.00580.2013 24585778 PMC3989756

[B56] Xu S. White H. D. Offer G. W. Yu L. C. (2009). Stabilization of helical order in the thick filaments by blebbistatin: further evidence of coexisting multiple conformations of myosin. *Biophys. J.* 96 3673–3681. 10.1016/j.bpj.2009.01.049 19413972 PMC2711421

[B57] Yu L. C. Brenner B. (1989). Structures of actomyosin crossbridges in relaxed and rigor muscle fibers. *Biophys. J.* 55 441–453. 10.1016/s0006-3495(89)82838-3 2930830 PMC1330498

[B58] Zhang X. Kampourakis T. Yan Z. Sevrieva I. Irving M. Sun Y. B. (2017). Distinct contributions of the thin and thick filaments to length-dependent activation in heart muscle. *elife* 6:e24081. 10.7554/eLife.24081 28229860 PMC5365314

[B59] Zhao F. Q. Padron R. Craig R. (2008). Blebbistatin stabilizes the helical order of myosin filaments by promoting the switch 2 closed state. *Biophys. J.* 95 3322–3329. 10.1529/biophysj.108.137067 18599626 PMC2547462

