# Supplementary material for: Cycling Cross-Bridges Contribute to Thin Filament Activation in Human Slow-Twitch Fibers
Source: Front Physiol. 2020 Mar 24;11:144. doi: 10.3389/fphys.2020.00144 (PMC7105683; doi:10.3389/fphys.2020.00144)
Supplement: Supplementary file 1 [file Data_Sheet_1.pdf]

Supplementary Material

METHODS

Quantification of the amount of troponin exchange by PAGE-Western blot analysis

Single fast-twitch fibers isolated from rabbit psoas muscles bundles (fast-twitch control containing fsTn), single slow-twitch fibers isolated from human soleus muscles bundles (slow-twitch control containing ssTn) and single slow-twitch fibers isolated from human soleus muscles bundles which were used for the experiments (slow-twitch, in which exogenous fsTn-IANBD was exchanged for endogenous ssTn) were suspended for 10 min at room temperature in 8 µL RotiLoad lysis buffer (Carl Roth GmbH. Karlsruhe, Germany) and then heated for 4 min to 80°C. Thereafter, the troponin I subunits were separated by 1-D sodium dodecyl sulfate polyacrylamide gel electrophoresis (SDS-PAGE) run in a custom-made device (12 % separation gel, 3 % stacking gel, 3 h. at 20 mA). The separated troponin I subunits were transferred to a nitrocellulose-membrane (0.22 µm pore size. GE Healthcare Europe GmbH, Freiburg, Germany) with a wet tank blotting system by constant 30 V for 1.5 hours at room temperature (Mini-Trans-Blot-Cell. Bio-Rad Laboratories GmbH. München, Germany). Membranes were subsequently washed (2x20 min., TBS), blocked overnight (3% non-fat milk powder) and then incubated with monoclonal anti-troponin I antibody (H86550 [AA186-192]. Meridian Life Sciences Inc. Memphis, Tennessee, USA). After washing and incubating with the second antibody (Goat Anti-Mouse IgG (H+L)-HRP. 172-1011, BioRad. Bio-Rad Laboratories GmbH. München, Germany) the skeletal troponin I bands were visualized using enhanced chemiluminescence (ImageQuant LAS 4000. GE Health Care Europe GmbH. Freiburg, Germany). The relative content of fast-twitch and slow-twitch skeletal troponin I was calculated with specialized software (TotalLab TL100, Nonlinear Dynamics. Newcastle, UK).

Experimental solutions

All solutions were adjusted to pH 7.0 at the experimental temperature (20°C). All concentrations are given in mM except when noted otherwise. Pre rigor solution contained 10 imidazole, 2.5 EGTA, 7.5 EDTA and 135 potassium propionate. Rigor solution contained 10 imidazole, 2.5 EGTA, 2.5 EDTA and 150 potassium propionate. Relax and activating solutions contained 10 imidazole, 2.0 $MgCl_2$, 1.0 MgATP, 1.0 CaEGTA, 50 sodium creatine phosphate and 500 U/mL of creatine kinase. Solutions with different pCa were obtained by mixing appropriated volumes of relaxing and activating solutions. The exchange buffer contained 20 MOPS, 5 $MgCl_2$, 5 EGTA 240 KCl, 5 DTT and a standard protease inhibitor cocktail. Chemicals were obtained from Sigma-Aldrich Chemie GmbH. Munich, Germany.

RESULTS

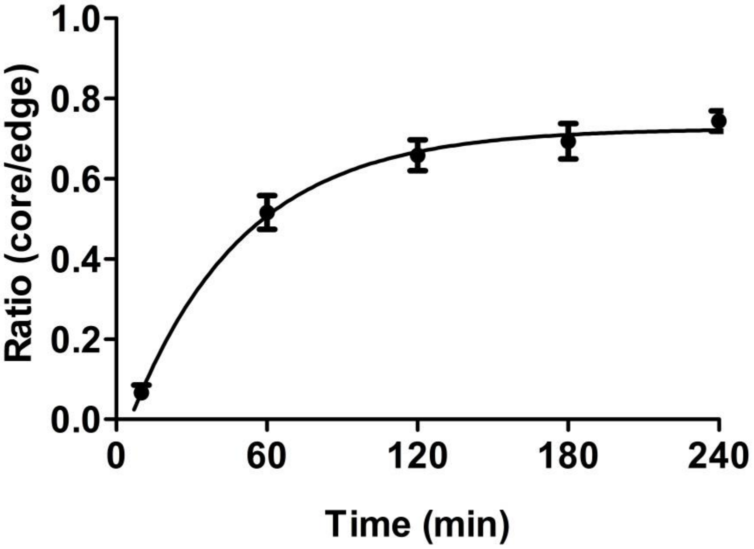

Supplementary figure 1. Core/edge ratio of the mean intensity profiles at different incubation times (see insets in Fig. 1 A). After 180 min the relative increase of the fluorescence at the central core becomes asymptotic. n = 6.

Supplementary Table 1. Effects of fsTn-IANBD exchange and mechanical experiments on functional properties of exchanged fibers.

	After mounting		After fsTn-IANBD exchange		After pCa-Force	After AmBleb
	Human soleus slow-twitch	Rabbit psoas fast-twitch	Human soleus slow-twitch	Rabbit psoas fast-twitch	Human soleus slow-twitch	
Force at pCa 4.5	100	100	92 ±3.9	87 ±3.6	83 ±3.6	6.1 ±0.8
Force at pCa 7.5	1.62 ±0.27	1.09 ±0.41	3.70 ±1.07	1.64±0.32	3.45±0.76	1.14±0.35
k_{tr} at pCa 4.5	100	100	78 ±8.1	83 ±7.0	68 ±6.0	

For every variable values are normalized to the values obtained at pCa 4.5 after mounting the respective fiber type.

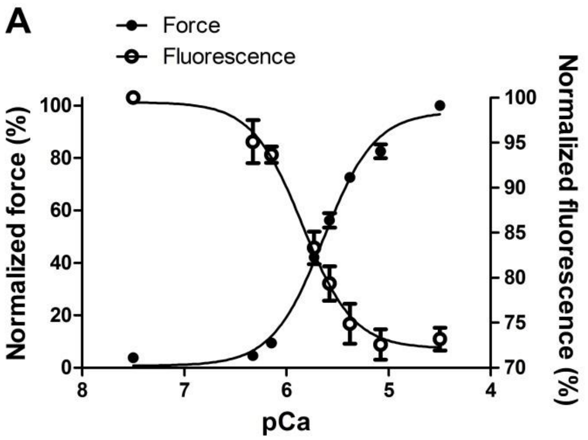

Supplementary Figure 2. Direct comparisons of steady state force and thin filament activation before (A) and after (B) incubation in 50 µM AmBleb. Note the decreased force development and thin filament activation (as shown by the change in fluorescence emission) in B.
